# Application of a 360-Degree Radiation Thermosonication Technology for the Inactivation of *Staphylococcus aureus* in Milk

**DOI:** 10.3389/fmicb.2021.771770

**Published:** 2021-11-05

**Authors:** Jianwei Zhou, Lele Sheng, Ruiling Lv, Donghong Liu, Tian Ding, Xinyu Liao

**Affiliations:** ^1^School of Mechanical and Energy Engineering, Ningbotech University, Ningbo, China; ^2^Department of Food Science and Nutrition, Zhejiang University, Hangzhou, China; ^3^Ningbo Research Institute, Zhejiang University, Ningbo, China

**Keywords:** 360-degree radiation thermosonication, inactivation kinetics, *Staphylococcus aureus*, quality characteristic, milk

## Abstract

Milk is easy to be contaminated by microorganisms due to its abundant nutrients. In this study, a 360-degree radiation thermosonication (TS) system was developed and utilized for the inactivation of *Staphylococcus aureus* in milk. The 360-degree radiation TS system-induced inactivation kinetics of *S. aureus* was fitted best by the Weibull model compared with biphasic and linear models. The treatment time, the exposure temperature, and the applied ultrasound power was found to affect the bactericidal efficacy of the 360-degree radiation TS system. Additionally, the TS condition of 200 W and 63°C for 7.5 min was successfully applied to achieve complete microbial inactivation (under the limit of detection value) in raw milk. The treatment of 360-degree radiation TS can enhance the zeta potential and decrease the average particle size of milk. It also exhibited better retainment of the proteins in milk compared with the ultrahigh temperature and conventional pasteurization processing. Therefore, the 360-degree radiation TS system developed in this study can be used as an alternative technology to assure the microbiological safety and retain the quality of milk, and the Weibull model could be applied for the prediction of the inactivation levels after exposure to this technology.

## Introduction

Milk contains a variety of nutritional compounds, especially high-quality proteins, making it one of the most popular food for human (Vartanian et al., 2007). In 2019, the global production of raw milk reached a value of 883.2 million tons (FAO, 2019). However, milk is also an ideal medium for the proliferation of pathogens, such as *Escherichia coli*, *Salmonella Thyphimurium*, and *Staphylococcus aureus*, making it a potential risk to food safety (Claeys et al., 2013; Zeinhom and Abdel-Latef, 2014; Rainard et al., 2018; Balasubramanian et al., 2019). Therefore, it is essential to control microbial contamination in the milk production process. Thermal treatments are the most widely used methods for the disinfection of milk, such as pasteurization and ultrahigh temperature processing (UHT) (Grant et al., 1996, 1998). However, heat may disrupt the thermosensitive nutrients in milk, which makes it difficult to meet the increasing nutrient requirements of consumers. Therefore, non-thermal processing technologies have emerged as alternatives to thermal methods for milk processing (Aneja et al., 2014; Song et al., 2016; Liao et al., 2018; Makroo et al., 2020).

Ultrasound, as a promising non-thermal technology, has attracted much attention because of its low working temperature and maximum retainment of food quality (Tao and Sun, 2015). The phenomenon of acoustic cavitation is considered as the major contributor to the microbial inactivation induced by ultrasound exposure. When cavitation bubbles collapse in the liquid medium, both mechanical (e.g., shock waves, liquid microjets, and shear forces) and sonochemical (formation of H⋅, OH⋅, and H_2_O_2_) effects will be acted on the microbial cells (Ashokkumar, 2011; Chemat et al., 2011; Awad et al., 2012). However, the antimicrobial efficiency of individual ultrasound treatment was limited (Li et al., 2016). Ultrasound combined with mild heat as a novel hurdle technology, also called thermosonication (TS), is of great interest because of efficient microbial inactivation and less damages in food quality (Barba et al., 2017).

Horn probes (also called sonotrodes) are the most commonly used devices for the generation of ultrasound. To use, horn probes, with the diameter ranging 2–12.7 mm, are dipped into the liquid sample. For most of the horn probes, nearly all the ultrasonic energy is transmitted through the tip of the probe with a small area (Campoli et al., 2018; Carrillo-Lopez et al., 2020; Yuksel and Elgun, 2020). Generally, horn probe-based devices are suitable for the treatment of samples with a small volume in the laboratory scale. However, when scaling up to industrial application, the efficacy of the conventional horn probes might be compromised by the uneven radiation of the acoustic field and low ultrasound energy intensity applied in the treated samples with large volumes (Abesinghe et al., 2019).

The objectives of this study were to develop a large-volume 360-degree radiation TS system and to apply this technology for inactivating *S. aureus* in milk, one of the foodborne pathogens of concern in milk safety. The microbial inactivation efficacy of this novel TS treatment was estimated and compared with conventional UHT and pasteurization processing. The 360-degree radiation TS-induced killing curve of *S. aureus* was fitted with mathematical models to describe the inactivation kinetics. Additionally, the effect of this TS treatment on the physicochemical characteristics of milk was evaluated.

## Materials and Methods

### Preparation of Bacterial Suspensions

*Staphylococcus aureus* strains of JPHG05A009-17, JP-GX07, and 5JP-HBA 2020 isolated from milk or milk product in China were used in this study. Bacterial culture was stored at −80°C in a mixture of glycerol and nutrient broth (NB) (Hope Bio-technology Co., Ltd., Qingdao, China) at a ratio of 1:1. Each strain was streaked and maintained on the Baird Parker (BP) (Hope Bio-technology Co., Ltd., Qingdao, China) medium supplemented with egg yolk tellurite emulsion (Hope Bio-technology Co., Ltd., Qingdao, China). A single colony of each strain was transferred into 100-ml NB, followed by incubation in a reciprocal shaker at 150 rpm and 37°C for 18 h. Subsequently, centrifugation (2,320 × *g*, 4°C, 10 min) was performed to collect *S. aureus* cells, which were washed twice by resuspension in sterile phosphate buffer solution (PBS) (pH 7.4). The suspensions of three *S. aureus* strains with the same volume were mixed thoroughly to prepare a cocktail inoculum, and the final concentration of the bacterial inoculum was approximately 10^9^ CFU/ml, with determination through plating on the plate count agar.

### Milk Preparation

Sterilized milk sample was purchased from a local market in Hangzhou, China. Raw milk without pasteurization was obtained from a farm in Hangzhou, China, and transported to the laboratory as soon as possible (less than 30 min). Milk samples with a volume of 270 ml were inoculated by *S. aureus* cocktail suspension (30 ml) to achieve the final bacterial concentration of approximately 10^8^ CFU/ml in milk.

### Thermosonication Treatment Conditions

**FIGURE 1:**
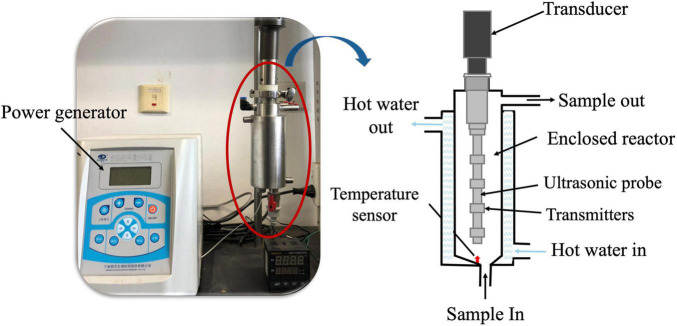
Diagram of the 360-degree radiation thermosonication system.

In this study, a 360-degree radiation TS equipment (Ningbo Scientz Co., Ltd., Ningbo, China) was employed to treat the inoculated milk samples and consisted of an electrical power generator, an enclosed cylinder-shaped reactor, a circulating water bath, a transducer, and an ultrasonic probe (Figure 1). The sample reactor is an enclosed chamber covered with a heating jacket chamber, which is equipped with a heating water outlet and inlet and a temperature sensor. There are multiple transmitters distributed on the side surface of the ultrasonic probe, which can emit a more uniform sonication field throughout the liquid sample. All the ultrasonic transmitters are controlled by one ultrasonic transducer, which is fixed on the top of the enclosed chamber and connected with the ultrasonic probe. The transducer comprises a cylindrical barrel, of which a shell is made of stainless steel, and the multiple ultrasonic transduction components are installed in an inner cavity of the barrel along the axis. Each ultrasonic transduction component comprises a supporting block, wherein over two piezoelectric ceramic piece groups are arranged on the sides of each supporting block. Each piezoelectric ceramic piece group consists of over two piezoelectric ceramic wafers, and the electrodes are arranged on the end faces of the piezoelectric ceramic wafers. The piezoelectric ceramic piece groups are fixed with the supporting blocks through pressing the blocks correspondingly, and the ultrasonic transduction components are closely connected with the inner wall of the cylindrical barrel. It can prevent the dropping of the electrodes and the deformation fracture of the piezoelectric ceramics. Additionally, the transducer has the capability of emitting even ultrasonic within the radial direction range of 360-degrees, and a higher transduction efficiency can be achieved.

The milk sample was placed in the cylinder-shaped reactor with a volume of 300 ml and a diameter of 5 cm. The ultrasonic probe has a length of 8 cm and a diameter of 1.2 cm. Four transmitters are longitudinally distributed on the side surface of the ultrasonic probe at an interval of 3.5 cm. The ultrasonic frequency is 20 kHz, and the maximum input ultrasonic power is 900 W. The samples treated by TS conditions were divided into four groups: 200 or 400 W at 55°C for 30 min and 200 or 400 W at 63°C for 15 min. The treatment temperature is achieved by the circulating water bath and controlled by a temperature sensor. The sample without TS treatment was set as the control.

### Pasteurization and Ultrahigh Temperature Treatment

The pasteurization of the raw milk samples (300 ml) was performed by the 360-degree radiation TS equipment with an ultrasonic power of 0 W. The treatment temperature was set at 63°C and maintained for 30 min. Regarding UHT treatment, a commercial UHT system (TG-UHT-CH-DJ-nQJ, Shanghai Nanhua Transducer Manufacture Co., Ltd., Shanghai, China) was used for treating the raw milk samples at 121°C for 15 s.

### Microbiological Analysis

Plate count method was used for the microbiological analysis. After various treatments, the milk samples were serially diluted with sterile 0.85% (m/v) saline solution. Then, a portion of diluent (0.1 ml) was spread on the BP medium supplemented with egg yolk tellurite emulsion. The plates were subsequently incubated in an incubator at 37°C for 48 h. The bacterial colonies grown on media was enumerated and expressed in log_10_ CFU/ml.

### Establishment of the Inactivation Kinetic Model

The inactivation of *S. aureus* in milk samples by a 360-degree radiation TS was described using the Weibull model (Eq. 1), biphasic model (Eq. 2), and linear model (Eq. 3) (Chen et al., 2019; Shao et al., 2020; Zhao et al., 2020). The data were analyzed and fitted by non-linear least squares regression method.


(1) $$l\,o\,g\,\left(\frac{N_{t}}{N_{0}}\right)=-\frac{1}{2.303}\,{\left(\frac{t}{a}\right)}^{b}$$

where *N*_0_ is the initial counts of *S. aureus*, *N*_*t*_ is the survival of *S. aureus* at treatment time *t*, *b* represents the time scale parameter, and *a* is a dimensionless shape parameter.


(2) $$l\,o\,g\,\left(\frac{N_{t}}{N_{0}}\right)=l\,o\,g\,\left[f\,e^{-P\,t}+\left(1-f\right)\,e^{-Q\,t}\right]$$

where *N*_0_ is the initial counts of *S. aureus*, *N*_*t*_ is the survival of *S. aureus* at treatment time *t*, *P* and *Q* represent the inactivated rates for two phases, and *f* and (1-*f*) are the ratios of TS-resistant and -susceptible *S. aureus* subpopulations, respectively.


(3) $$l\,o\,g\,\left(\frac{N_{t}}{N_{0}}\right)=a\,t+b$$

where *N*_0_ is the initial count of *S. aureus*, *N*_*t*_ is the survival of *S. aureus* at treatment time *t*, and *a* represents the inactivated rate.

The fitness of models was estimated with the following goodness-of-fit indexes: the coefficient of determination (*R*^2^, Eq. 4) and the root mean square error (RMSE, Eq. 5).


(4) $$R^{2}=1-\frac{S\,S_{r}}{S\,S_{t}}$$

where SS represents the residual sum of squares, and SS_*t*_ is the total sum of squares.


(5) $$R\,M\,S\,E=\sqrt{\frac{\sum_{i=1}^{n}{\left(y_{o\,b\,s}-y_{p\,r\,e\,d}\right)}^{2}}{n}}$$

where the variable *y*_*obs*_ was the logarithm value of *S. aureus* population estimated by the plate count method (log_10_ CFU/ml) in this study, *y*_*pred*_ corresponds to the logarithm value of the bacterial population (log_10_ CFU/ml) calculated by the fitted model, *n* is the total number of the observed data points, and *p* was the number of parameters for the estimated model.

### Determination of the Physicochemical Characteristics of Milk

#### Color Properties

The color properties of milk samples after various treatments were measured using a Konica Minolta CM-600d spectrophotometer (Konica Minolta Holdings, Inc., Tokyo, Japan) in the reflection mode at room temperature (25 ± 1°C). The parameters of L* (lightness), a* (redness and greenness), and b* (yellowness and blueness) were obtained for calculation of the total color difference Δ*E* by Eq. 6 (Zhao et al., 2019; Feng et al., 2020):


(6) $$△\,E=\sqrt{{\left(L^{*}-L_{o}^{*}\right)}^{2}+{\left(a^{*}-a_{o}^{*}\right)}^{2}+{\left(b^{*}-b_{o}^{*}\right)}^{2}}$$

where *L*_0_, *a*_0_, and *b*_0_ represented the color values of the milk sample before treatments.

#### pH

The pH of the milk samples was measured with a digital pH meter PHS-550 (Lohand Biological Co., Ltd., Hangzhou, China) at room temperature (25 ± 1°C).

#### Particle Size and Zeta Potential

After the different treatments, the particle size and zeta potential of the milk samples were determined using the Malvern Zetasizer Nano (ZS90; Malvern Panalytical Ltd., Malvern, United Kingdom) at room temperature (25 ± 1°C).

#### Total Protein Content

The total protein content (TPC) in the milk samples was measured according to the Kjeldahl method. Copper sulfate (0.4 g), potassium sulfate (6 g), and 20 ml sulfuric acid were added to a portion of milk sample (10 g) for the digestion to convert all the organically bonded nitrogen into ammonium ions. Subsequently, the ammonium ions are reacted with sodium hydroxide and transformed into ammonia, which dissolve in boric acid. Finally, the standard solution of hydrochloric acid (HCl) was used for titration to determine the nitrogen concentration. TPC (g/100 g) was calculated by the following equation:


(7) $$x=\frac{\left(V_{1}-V_{0}\right)\times c\times 0.0140}{m\times V_{2}/100}\times F\times 100$$

where *V*_1_ corresponds to the consumed volume (ml) of HCl standard titrant with concentration of *c* (M), *V*_0_ is the consumed volume (ml) of HCl used for the blank sample (water), *m* was the quantity of the samples (g), *V*_2_ represents the volume of the digested solution (ml), and *F* is the nitrogen-to-protein conversion coefficient, which is 6.38 for milk.

### Statistical Analysis

All experiments in this study were performed in triplicate. Determining the statistical significance of the data was carried out using SPSS 22.0 (IBM Corp., Armonk, NY, United States) using one-way analysis of variance with Duncan’s test for the *post-hoc* multiple comparison. A value of *p* < 0.05 was considered statistically different. The kinetic models were fitted to the data points with Origin 8.0 (OriginLab Corp., Northampton, MA, United States).

## Results and Discussion

### 360-Degree Radiation Thermosonication-Induced Heat Transfer Enhancement in Milk

**FIGURE 2:**
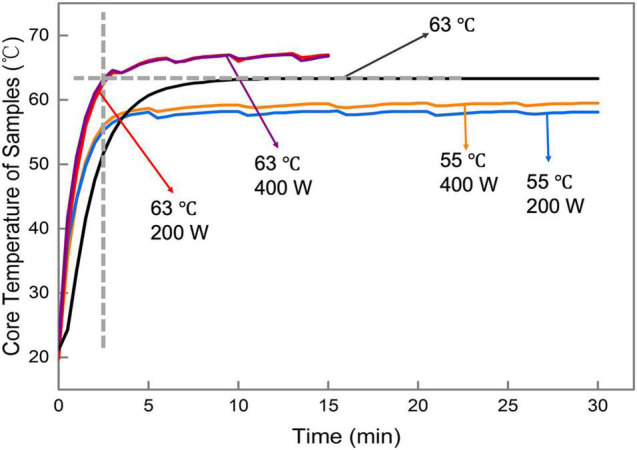
Core temperature changes of samples under different treatment conditions. The black line shows the temperature change without ultrasonic treatment.

During the 360-degree radiation TS treatment, the milk temperatures are controlled by circulating water in the jacket chamber covering the side surface of the sample reactor (Figure 1). As shown in Figure 2, the core temperature of the milk samples was increased to the target temperatures (55 or 63°C) in 5 min, and it was maintained at a relatively constant value with a deviation of less than 3.33°C from the target temperature. It demonstrated that the 360-degree radiation TS system could efficiently inhibit the perturbation of temperature. Without the ultrasonic effect, it required 18 min for the temperature in the core of milk sample to reach the target pasteurization temperature of 63°C. It might be attributed to the effect of acoustic cavitation during the ultrasound processing, which could accelerate the heat transfer efficiency between the milk samples in the reactor and the heating water in the jacket chamber (Kurbanov and Melkumov, 2003; Legay et al., 2011). When cavitation bubbles collapse, a large amount of energy can be generated, and violent impact and high-speed micro-jet are also formed, which greatly enhance the collision density. The thermal and velocity boundary layers are subsequently disrupted, which decreases the thermal resistance and creates microturbulence to achieve the heat transfer enhancement.

### Inactivation of *S. aureus* Cocktail in Milk by 360-Degree Radiation Thermosonication

The survival levels of *S. aureus* cocktail in milk treated by the 360-degree radiation TS treatment are exhibited in Table 1. The 360-degree radiation TS-induced inactivation of *S. aureus* was affected by the treatment time, the exposure temperature, and the applied ultrasound power.

**TABLE 1:** Survival concentrations (log_10_ CFU/ml) of *S. aureus* after the 360-degree radiation thermosonication treatment under various conditions.

**Treatment time (min)**	**Exposure temperature (°C)/Applied ultrasonic power (W)**			
	**55/200**	**55/400**	**63/200**	**63/400**
3	7.46 ± 0.50C,b	6.90 ± 0.59D,b	4.79 ± 0.20E,a	4.68 ± 0.41D,a
6	5.89 ± 0.32B,b	5.47 ± 0.33C,b	3.62 ± 0.33D,a	3.79 ± 0.61C,a
9	5.38 ± 0.54B,c	4.60 ± 0.19B,b	2.65 ± 0.22C,a	3.02 ± 0.12B,a
12	5.00 ± 0.79B,C,c	4.10 ± 0.17AB,b	1.92 ± 0.17B,a	1.99 ± 0.24A,a
15	4.26 ± 0.28A,c	3.93 ± 0.16A,b	1.33 ± 0.17A,a	1.59 ± 0.22A,a

*The initial concentration of bacterial cells was about 8.12 ± 0.05 log_10_ CFU/ml. The values are mean of triplicate measurements ± standard deviation. The values with different lowercase letters in the same row and uppercase letters in the same column showed a significant difference at *p* < 0.05.*

As shown in Table 1, there is an inverse correlation between the *S. aureus* survival population and the treatment time or exposure temperature. With TS treatment at 63°C and 400 W, the survival levels of *S. aureus* were 8.12, 4.68, 3.79, 3.02, 1.99, and 1.59 log for 0-, 3-, 6-, 9-, 12-, and 15-min treatment time, respectively. When the exposure temperature decreased from 63 to 55°C, the remaining survival level increased from 1.59 to 3.93 logs for a treatment time of 15 min. The effect of ultrasound powers on TS-induced bactericidal efficacy is closely related to the exposure temperature. Under 55°C, the enhancement of ultrasound power contributed to the higher reduction of *S. aureus* in milk to some extent. When the exposure time was 15 min, the *S. aureus* cells were decreased by 3.86 log under an applied ultrasound power of 200 W, and it was 4.19 log reduction when the ultrasound power was increased to 400 W. However, when exposing the milk samples to 63°C, the survival levels of *S. aureus* were independent of ultrasound powers (*p* > 0.05).

Most of the commonly used ultrasonic horns have only one transmitter on the tip, which transfers nearly all the energy through one direction. In our previous study, it was found that the TS, combined with single-direction ultrasonic radiation (600 W) and 63°C, resulted in less than 99% reduction of *S. aureus* for 5 min (Li et al., 2019). Individual heat exposure has been reported to activate protective systems in bacteria cells, which could compromise the microbial inactivation level (Liu et al., 2020; Wu et al., 2021). In combination with ultrasonic field, the effect of ultrasound-induced cavitation contributes to the rupture of the microbial outer structure (e.g., cell wall/membranes), which weakened the heat tolerance of microorganisms and led to final cell death.

The 360-degree radiation TS treatment in this work under an applied ultrasound power of 200 W and a temperature of 63°C brought in over 99.9% inactivated level of *S. aureus* after 3 min of exposure. According to the performance criteria of milk processing published by FAO/WHO, the processing treatment should be designed to achieve at least a 5-log reduction of the target bacteria. In this study, a TS treatment of 63°C and 200/400 W for 9 min resulted in over 5-log reduction of *S. aureus*, while it required 40 min for an individual thermal treatment at 63°C to decrease *S. aureus* by over 5 logs. Considering the bactericidal efficacy and the energy consumption (the applied ultrasound power), the 360-degree radiation TS treatment of 63°C and 200 W was selected for further analysis in this study.

### Establishment of Inactivation Kinetics

In order to predict the behavior of *S. aureus* cells in milk when exposed to the 360-degree TS treatment, the survival curve was fitted to linear and non-linear (Weibull and biphasic) models. *R*^2^ and RMSE are the most commonly used indexes for the estimation of the goodness-of-fit of the models. A *R*^2^ value close to 1 and a low RMSE value indicate that the fitted model exhibits a good fitting to data points.

As shown in Table 2, the values of *R*^2^ and RMSE of the linear model are 0.866 and 76.08, respectively, which demonstrated that the inactivation kinetics of *S. aureus* by the 360-degree radiation TS treatment did not follow a linear pattern. Regarding non-linear models, the obtained values of *R*^2^ for the fitted biphasic model and Weibull model are 0.985 and 0.999, respectively, which are close to 1. However, the value of RMSE of the fitted biphasic model is 6.33, which is much higher than that (0.36) of the fitted Weibull model in this study. Overall, the Weibull model is the most suitable model to describe the 360-degree radiation TS-induced inactivation kinetics of *S. aureus* in milk. Similarly, non-linear curves of TS-induced microbial inactivation have been observed in previous studies (Lee et al., 2009). In the study of Sasikumar et al. (2019), it was found that the inactivation level of *E. coli* by TS (1.95 W/50°C/21 min) in khoonphal juice was fitted well with the Weibull model, with an *R*^2^ value of over 0.99 (Sasikumar et al., 2019).

**TABLE 2:** The fitted parameters of linear model, biphasic model, and Weibull model.

**Linear model**				**Biphasic model**					**Weibull model**			
***A***	***B***	***R* ^2^**	**RMSE**	***f***	***p***	***q***	***R* ^2^**	**RMSE**	***a***	***b***	***R* ^2^**	**RMSE**
−1.39 ± 0.76	−0.38 ± 0.07	0.866	76.08	0.996 ± 0.001	90091.86 ± 0.00	0.68 ± 0.17	0.985	6.33	0.02 1 ± 0.004	0.416 ± 0.001	0.999	0.36

The Weibull model is built by two parameters, including the scale parameter *a* (time) and the dimensionless shape parameter *b*. The shape parameter *b* not only accounts for the concavity of a survival curve but also is related to the physiological states of microorganisms (Surowsky et al., 2014). *b* < 1 indicates the upward concavity of a survival curve and that the remaining microbial subpopulation has the ability to adapt to the applied treatment, and *b* > 1 reflects the survival curve with a downward concavity and that the treatment results in accumulative damages on the remaining bacterial cells. As shown in Table 2, the *b* value of the fitted Weibull model in this work is 0.42, less than 1, indicating that the remaining *S. aureus* cells become increasingly damaged by the 360-degree radiation TS exposure. Based on the established Weibull model, the time to achieve a 5-log reduction is calculated to be 7.5 min. Therefore, the conditions of the 360-degree radiation TS treatment for the sterilization of milk are 200 W at 63°C for 7.5 min.

### Application of 360-Degree Radiation Thermosonication for the Sterilization of Raw Milk

In order to validate the application of the 360-degree radiation TS treatment, it was further used for the sterilization of raw milk in comparison to commercial UHT processing (121°C, 15 s) and pasteurization (63°C, 30 min). As shown in Table 3, the initial bacterial contamination level in raw milk was 3.25 log_10_ CFU/ml. After all the treatments, the bacterial concentrations were decreased to be under the limit of detection (LOD), indicating that the 360-degree radiation TS treatment (200 W, 63°C, 7.5 min) in this work can be used as a promising technology for assurance of the microbiological safety of milk.

**TABLE 3:** The total viable counts in milk before and after various treatments.

**Treatments**	**Total viable counts (log_10_ CFU/ml)**
Raw milk	3.25 ± 0.77
360-degree radiation thermosonication (200 W, 63°C, 7.5 min)	ND
UHT (121°C, 15 s)	ND
Pasteurization (63°C, 30 min)	ND

*ND, non-detected (i.e., below the limit of detection).*

### Physicochemical Property Changes of Raw Milk

#### Changes in pH, Particle Size, and Zeta Potential

The pH values of milk are stability indices of the casein micelles. The pH value of the untreated raw milk is 6.65, and no significant changes (*p* > 0.05) in pH values were found in the milk samples treated with the 360-degree radiation TS, UHT, and pasteurization (Table 4).

**TABLE 4:** pH, zeta potential, and particle size of milk after various treatments.

**Treatments**	**pH**	**Zeta potential (mV)**	**Particle size (μm)**
Raw milk	6.65 ± 0.04a	−23.0 ± 1.6a	7.59 ± 0.16c
360-degree radiation thermosonication (200 W, 63°C, 7.5 min)	6.66 ± 0.02a	−37.0 ± 0.4d	4.62 ± 0.14a
UHT (121°C, 15 s)	6.69 ± 0.01a	−30.4 ± 1.2c	6.08 ± 0.06b
Pasteurization (63°C, 30 min)	6.71 ± 0.04a	−26.4 ± 0.7b	6.00 ± 0.08b

*Values with different lowercase letters in the same column showed a significant difference at *p* < 0.05.*

The zeta potential is an important index to reflect the stability of a colloidal system because it is generated from the interaction of all charged particles in the system (Shanmugam and Ashokkumar, 2014). As shown in Table 4, the zeta potential of raw milk was −23 mV, and it was significantly (*p* < 0.05) increased after various treatments. The milk treated with the 360-degree radiation TS exhibited the highest zeta potential, which might be attributed to the ultrasound-induced damage and interruption in the membrane of the fat globule, which could make the membrane more negatively charged and lead to an increase in zeta potential (Scudino et al., 2020). The enhancement of zeta potential caused by the 360-degree radiation treatment could improve the interaction of fat globules and protein micelles and thus strengthen the stability of milk (Shanmugam and Ashokkumar, 2014).

The average particle sizes of milk with various treatments are shown in Table 4. Untreated raw milk has an average particle size of 7.59 μm. It was found that all the treatments significantly reduced the particle size, and the A 360-degree radiation TS-treated milk exhibited the smallest particle size of milk with a value of 4.62 μm. The cavitation phenomenon was considered to be the major contributor to the reduction of the particle size of milk (Ertugay et al., 2004).

#### Changes in Color Characteristics

Color is one of the most important characteristics for milk sales, which is related to the dispersion of fat globules and casein micelles in the visible spectrum. The changes in the color characteristics of raw milk treated by 360-degree radiation TS, UHT, and pasteurization are shown in Table 5. Compared with the untreated raw milk, the *L*^∗^ values (lightness) of milk were significantly increased, while the *b*^∗^ values (yellow) of milk were significantly decreased after all the treatments. The more intense acoustic cavitation provides better homogenization of the milk due to the reduction of the fat globule size. Great changes in the size of the fat droplets are enough to change the light reflection (Owens et al., 2001). Regarding the *a*^∗^ value (green), it was slightly increased after 360-degree radiation TS treatment, while UHT treatment resulted in a decrease in *a*^∗^ value. Overall, the total color difference (Δ*E*) of 360-degree radiation TS-treated milk is 3.55, which exhibits an insignificant difference from that of UHT-treated milk and significantly higher than that of pasteurized milk.

**TABLE 5:** The color changes of milk before and after various treatments.

**Treatments**	L*	a*	b*	**Δ*E***
Raw milk	87.38 ± 0.23a*	−2.00 ± 0.06b	4.67 ± 0.15a	0
360-degree radiation thermosonication (200 W, 63°C, 7.5 min)	90.75 ± 0.42c	−2.25 ± 0.04a	3.61 ± 0.04b	3.55 ± 0.40a
UHT (121°C, 15 s)	89.39 ± 0.08b	−1.79 ± 0.05c	2.60 ± 0.42c	2.91 ± 0.26b
Pasteurization (63°C, 30 min)	89.25 ± 0.42b	−1.98 ± 0.01b	4.01 ± 0.06b	1.99 ± 0.41c

*Values with different lowercase letters in the same column showed a significant difference at *p* < 0.05. The asterisk is used to differentiate the CIELAB system from ANLAB.*

#### Changes in the Total Protein Content

Protein is the most important component in milk, but it is sensitive to heat and easy to be denaturized. As shown in Table 6, the initial TPC was 3.53% in untreated raw milk. Both 360-degree radiation TS and UHT resulted in no significant changes of the TPC in milk. However, the TPC of pasteurized milk was significantly decreased to 2.53% (*p* < 0.05), which might have resulted from the heat-induced denaturation of proteins.

**TABLE 6:** The total viable counts in milk before and after various treatments.

**Treatments**	**Protein content (%)**
Raw milk	3.28 ± 0.19b
360-degree radiation thermosonication (200 W, 63°C, 7.5 min)	3.22 ± 0.25b
UHT (121°C, 15 s)	3.02 ± 0.16b
Pasteurization (63°C, 30 min)	2.53 ± 0.38a

*Values with different lowercase letters in the same column showed a significant difference at *p* < 0.05.*

## Conclusion

In the present study, a 360-degree radiation TS system was developed for the sterilization of milk. Compared with conventional pasteurization (63°C), the 360-degree radiation TS system enhanced the heat transfer rate in milk by over two times. Based on the established Weibull inactivation model, the treatment duration for the 360-degree radiation TS system to bring out a 5-log reduction was 7.5 min, which was much shorter than that of the common TS treatment combining the single-direction radiation ultrasonic horn and heat. Additionally, in this work, the 360-degree radiation TS system was successfully applied to achieve complete microbial inactivation (under LOD value) in raw milk. Regarding the effect on the physiochemical properties of milk, it was found that the treatment of 360-degree radiation TS can enhance the zeta potential and decrease the average particle size of milk. It also exhibited better retention of the proteins in milk compared with the UHT and conventional pasteurization processing. The 360-degree radiation TS technology can be utilized as a promising technology to assure the microbiological safety and retain the quality of milk.

## Data Availability Statement

The original contributions presented in the study are included in the article/supplementary material, further inquiries can be directed to the corresponding author/s.

## Author Contributions

XL and JZ conceived and designed the experiments. XL wrote the first draft of the manuscript. JZ performed the research and conducted the data analysis. LS, RL, DL, and TD contributed to the literature search and in reviewing and finalizing the manuscript. All authors have read and approved the final manuscript.

## Conflict of Interest

The authors declare that the research was conducted in the absence of any commercial or financial relationships that could be construed as a potential conflict of interest.

## Publisher’s Note

All claims expressed in this article are solely those of the authors and do not necessarily represent those of their affiliated organizations, or those of the publisher, the editors and the reviewers. Any product that may be evaluated in this article, or claim that may be made by its manufacturer, is not guaranteed or endorsed by the publisher.
